# Supplementary material for: Formulation of Stable and Homogeneous Cell-Penetrating Peptide NF55 Nanoparticles for Efficient Gene Delivery In Vivo
Source: Mol Ther Nucleic Acids. 2017 Oct 20;10:28–35. doi: 10.1016/j.omtn.2017.10.011 (PMC5723355; doi:10.1016/j.omtn.2017.10.011)
Supplement: Document S1. Figures S1–S5 and Table S1 [file mmc1.pdf]

OMTN, Volume *10*

Supplemental Information

Formulation of Stable and Homogeneous Cell-Penetrating Peptide NF55 Nanoparticles for Efficient Gene Delivery *In Vivo*

Krista Freimann, Piret Arukuusk, Kaido Kurrikoff, Ly Pärnaste, Raivo Raid, Andres Piirsoo, Margus Pooga, and Ülo Langel

Supplementary information

Table S1. The mean hydrodynamic diameter of the differently formulated nanoparticles

Formulation of nanoparticles	Time, min	Hydrodynamic diameter, nm (Intensity)	pDI	Size distribution by volume (%)
Formulation 1	1-60	124.8 ± 1.6	0.142 ± 0.01	98.6
Formulation 2	1	112.4 ±6.55<br>423 ± 59.57<br>4295 ± 36.23	0.608 ± 0.024	35<br>40.3<br>24.6
Formulation 2 Filtrated 200 nm	1	108.4 ± 5.39	0.122 ± 0.014	100
Formulation 2 Filtrated 200 nm	60	115 ± 4.285	0.120 ± 0.017	100
CCC	1	85.11 ± 14.02<br>2082 ± 991.6	0.211 ± 0.009	97.3<br>2.7
CCC	60	77.8 ± 0.41<br>2055 ± 818.2	0.184 ± 0.023	87.5<br>12.5

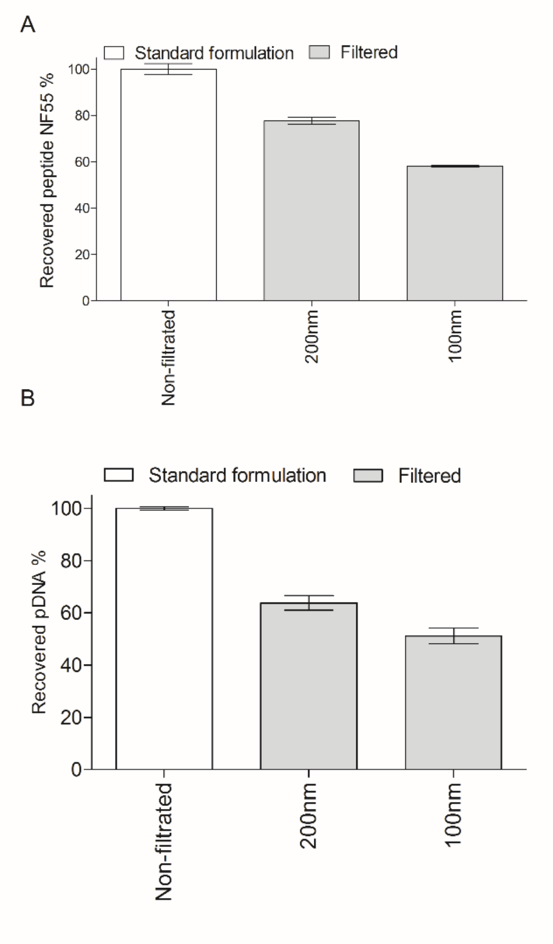

Figure S1. Recovery of peptide (A) and pDNA (B) after filtration of the formulated nanoparticles. PicoGreen (for pDNA) and the DC protein determination kit were used respectively to quantify the components of nanoparticles. The data are presented as the mean ± SEM (*n* = 3)

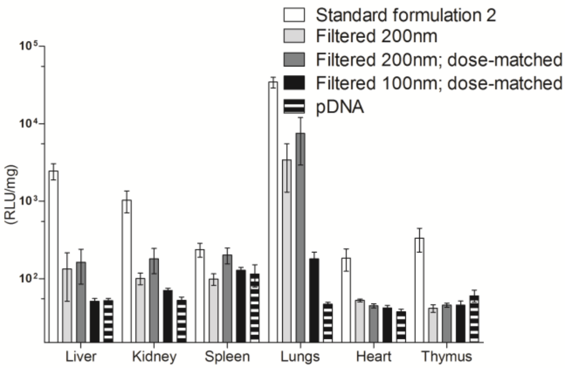

Figure S2. Expression of marker gene in Balb/c mice transfected by NF55/pDNA nanoparticles after filtration at CR4. The data are from at least five representative experiments, presented as the mean ± SEM

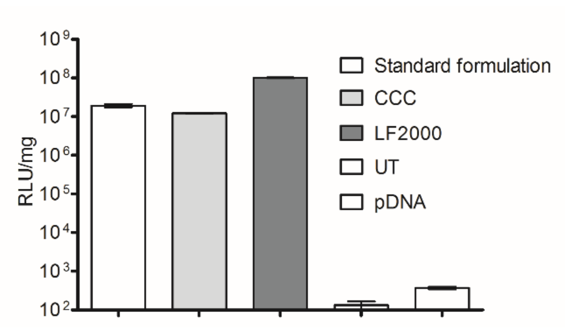

Figure S3. Expression of marker gene in CHO cells transfected by NF55/pDNA CCC nanoparticles at CR4. Untreated cells (UT) were used as a negative control and Lipofectamine™ 2000 (LF2000) was used as a positive control. The data are presented as the mean ± SEM (*n* = 3)

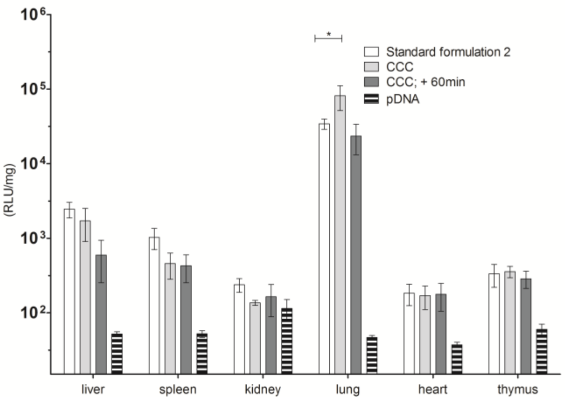

Figure S4. Expression of marker gene in Balb/c mice transfected by NF55/pDNA nanoparticles produced by CCC and standard formulation 2 at CR4. The data from at least five representative experiments are presented as the mean ± SEM, **P* < 0.05 (Student's *t*-test, two-tailed distribution, two-sample unequal variance).

A

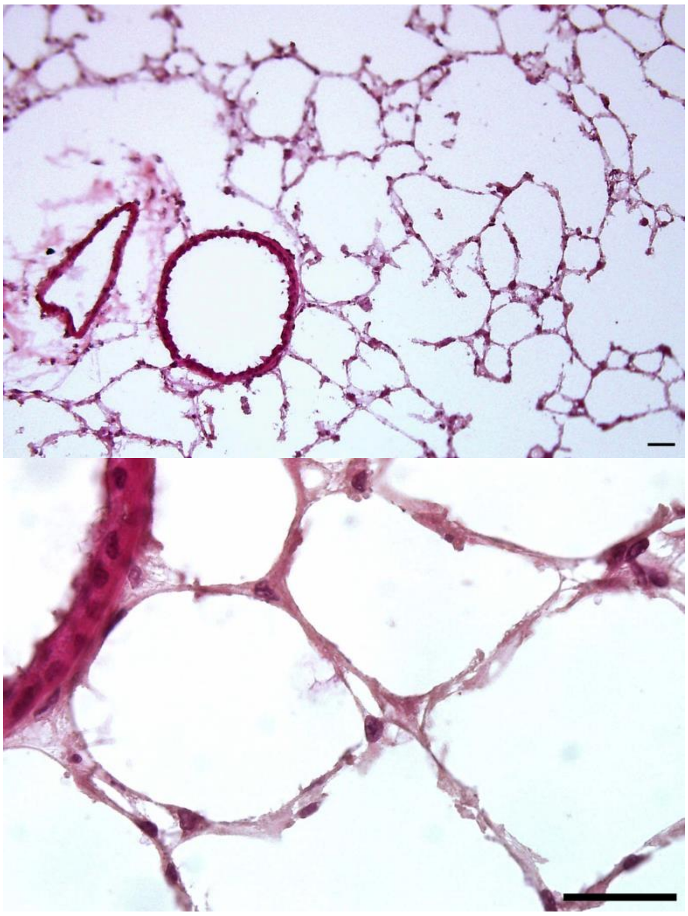

B

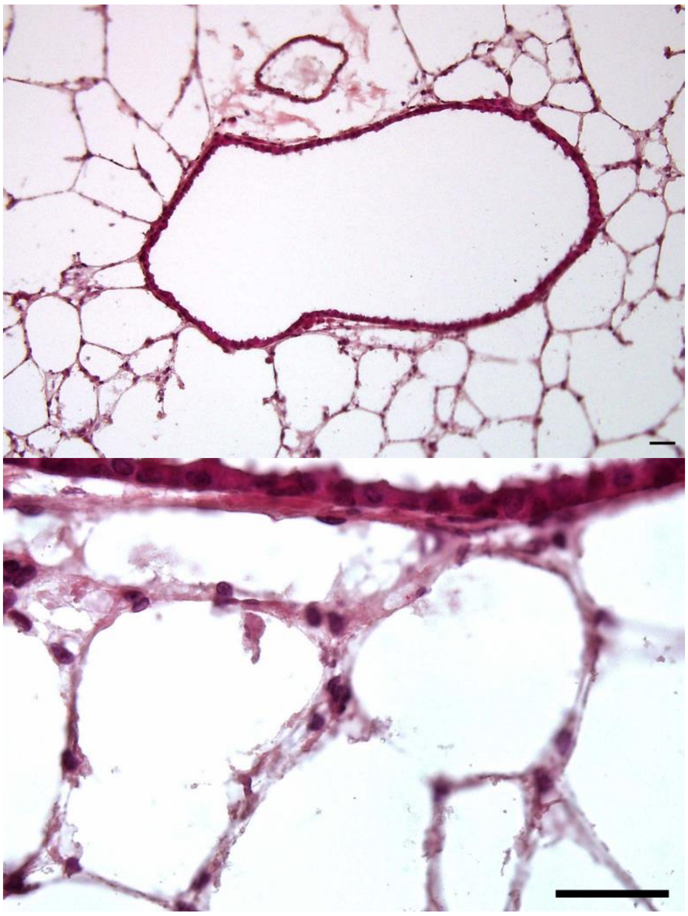

Figure S5. H&E stained tissue from representative lung areas of the animals that have received NF55/pDNA CCC (1 mg/kg pDNA dose) (A) or NF55/pDNA CCC with additional 60 min complex incubation (B). The size bar represents 30 μm.
