# Supplementary material for: Formulation of Stable and Homogeneous Cell-Penetrating Peptide NF55 Nanoparticles for Efficient Gene Delivery In Vivo
Source: Mol Ther Nucleic Acids. 2017 Oct 20;10:28–35. doi: 10.1016/j.omtn.2017.10.011 (PMC5723355; doi:10.1016/j.omtn.2017.10.011)
Supplement: Document S2. Article plus Supplemental Information [file mmc2.pdf]

Molecular Therapy
Nucleic Acids
Original Article

Formulation of Stable and Homogeneous Cell-Penetrating Peptide NF55 Nanoparticles for Efficient Gene Delivery *In Vivo*

Krista Freimann,[1] Piret Arukuusk,[1] Kaido Kurrikoff,[1] Ly Pärnaste,[1] Raivo Raid,[2] Andres Piirsoo,[4] Margus Pooga,[1,2] and Ülo Langel[1,3]

[1]Institute of Technology, University of Tartu, Nooruse, 50411 Tartu, Estonia; [2]Department of Developmental Biology, Institute of Molecular and Cell Biology, University of Tartu, Riia 23, 51010 Tartu, Estonia; [3]Department of Neurochemistry, The Arrhenius Laboratories for Natural Sciences, Stockholm University, 10691 Stockholm, Sweden; [4]Institute of Biomedicine and Translational Medicine, University of Tartu, Ravila 19, 50411 Tartu, Estonia

Although advances in genomics and experimental gene therapy have opened new possibilities for treating otherwise incurable diseases, the transduction of nucleic acids into the cells and delivery *in vivo* remain challenging. The high molecular weight and anionic nature of nucleic acids require their packing into nanoparticles for the delivery. The efficacy of nanoparticle drugs necessitates the high bioactivity of constituents, but their distribution in organisms is mostly governed by the physical properties of nanoparticles, and therefore, generation of stable particles with strictly defined characteristics is highly essential. Using previously designed efficient cell-penetrating peptide NF55, we searched for strategies enabling control over the nanoparticle formation and properties to further improve transfection efficacy. The size of the NF55/pDNA nanoparticles correlates with the concentration of its constituents at the beginning of assembly, but characteristics of nanoparticles measured by DLS do not reliably predict the applicability of particles in *in vivo* studies. We introduce a new formulation approach called cryo-concentration, where we acquired stable and homogeneous nanoparticles for administration *in vivo*. The cryo-concentrated NF55/pDNA nanoparticles exhibit several advantages over standard formulation: They have long shelf-life and do not aggregate after reconstitution, have excellent stability against enzymatic degradation, and show significantly higher bioactivity *in vivo*.

INTRODUCTION

Gene therapy applications and research on *in vivo* delivery vectors are among the most rapidly growing areas in nanomedicine research. Despite significant progress in the development of therapeutic nucleic acids, their widespread application is still held back by low cellular uptake due to very poor cell membrane permeability. Therefore, the development of safe, efficient, and clinically applicable gene delivery vectors are crucial for the clinical application of therapeutic nucleic acids.[1,2] *Cell-penetrating peptides* (CPPs) are short cationic and/or amphipathic peptides that have been applied for both the *in vitro* and *in vivo* delivery of various types of molecules, including small drug molecules, biomacromolecules like proteins, and nucleic acids.[3] Recently, we have designed highly promising transfection reagent NF55 for transduction of nucleic acid molecules into cells.[4] NF55 forms stable particles with nucleic acids and is an effective *in vivo* transfection reagent, enabling induction of gene expression with efficiency that is comparable to the best transfection reagents available at the moment.[4]

From a clinical point of view, it is highly essential that a pharmaceutical formulation to be administered has a strictly defined molecular composition and structural characteristics. One of the major problems with the nanoparticle formulation in the case of self-assembling systems is, in general, the heterogeneous size distribution and the difficulties with exact definition of drug composition.[5,6] Our CPP/DNA nanoparticle formulation approach of mixing DNA and peptide at appropriate ratios in water to form non-covalently coupled complexes has the clear advantage of using only two components with defined chemical composition compared to many other nanoparticles where more components are needed.[7] However, the problem of a heterogeneous size and its broad distribution of size might still persist with nanocomplexes assembled based on the electrostatic interactions.[8] Therefore, it is highly vital to prepare homogeneous, well-characterized particles for their further application *in vivo*, since the physiochemical parameters, including size and size distribution, influence both the biodistribution and toxicity of nanoparticles.[9] The nanoparticle size is one of major factors governing particle distribution *in vivo*. For example, it has been demonstrated that nanoparticles with diameters less than 5 nm rapidly undergo renal clearance upon intravenous administration.[9,10] The splenic filtration accounts for the retention of particles larger than 200 nm in diameter, and particles in the micrometer range (2–5 μm) have been shown to accumulate within capillaries of the lungs,[9–11] leaving a rather narrow-size

Received 19 June 2017; accepted 17 October 2017;
https://doi.org/10.1016/j.omtn.2017.10.011.

Correspondence: Krista Freimann, Laboratory of Molecular Biotechnology, Institute of Technology, University of Tartu, Nooruse 1, 50411 Tartu, Estonia.
E-mail: krista.freimann@ut.ee

 © 2017 The Authors.
This is an open access article under the CC BY license (http://creativecommons.org/licenses/by/4.0/).

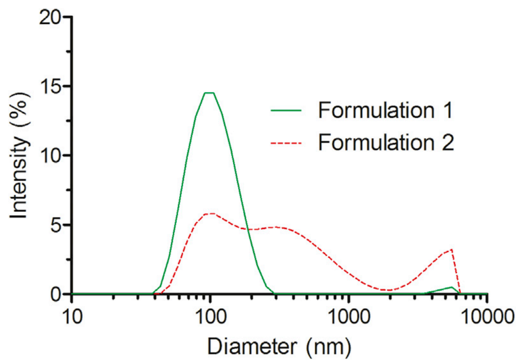

Figure 1. Size Distribution of Different Formulations of NF55/DNA Nanoparticles

Size distribution of NF55/DNA nanoparticles in formulation 1 (green line, peptide concentrations 40 μM for *in vitro* studies) and formulation 2 (dashed red line, 400 μM for *in vivo* studies). Data were analyzed using the Malvern Zetasizer.

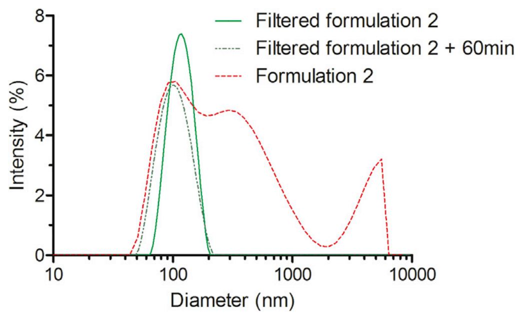

Figure 2. Size Distribution of NF55/DNA Nanoparticles after the Filtration of Formulation 2 Particles

The dashed red line represents the size distribution of NF55/DNA particles without filtration, and the green lines illustrate nanoparticles after filtration through the 200-nm filter. The dotted darker green line shows the size distribution of filtered nanoparticles after incubation for 60 min at room temperature. Data were analyzed using the Malvern Zetasizer.

window for nanoparticles that can circulate in organisms for longer to reach their target issue.

Thus, conglomeration and non-uniformity and broad size distribution most probably leads to stochastic and irreproducible distribution of nanoparticles in an organism, thereby decreasing the circulation time and diminishing the effect of active ingredient.[9] Therefore, the uniform size of nanoparticles, high homogeneity, and the lifetime are relevant topics to be addressed.

In the current study, we applied several approaches to tackle these issues and elaborated a new strategy for preparing homogeneous NF55/plasmid DNA (pDNA) nanoparticles for DNA delivery *in vivo*. Our strategy of producing cryo-concentrated formulations yielded non-aggregating nanoparticles with uniform size that are stable against enzymatic degradation and show significantly higher bioactivity *in vivo*.

RESULTS AND DISCUSSION

Nanoparticles Formed at a Higher Concentration Tend to Conglomerate

We have previously designed a highly efficient CPP for transduction of nucleic acids into cells and characterized the properties of electrostatically assembled NF55/pDNA nanoparticles and demonstrated their applicability for gene therapeutic use.[4] During characterization of NF55/pDNA nanoparticles, we discovered that in addition to major fraction of small separate NF55/pDNA nanoparticles, some of these had associated with each other and formed larger conglomerates/aggregates (typically d $\leq$ 300 nm).[4]

In order to further examine whether this conglomeration increases we assembled the nanoparticles at the 10-fold higher peptide and DNA concentration that are necessary for formulation of complexes for *in vivo* use (formulation 2). Indeed, aggregate formation was even more characteristic for the "*in vivo* formulation" procedure (Figure 1). Probably this was due to the higher concentration of components (400-μM peptide concentration, formulation 2) compared to the "*in vitro* studies" particles (40-μM peptide concentration, formulation 1), even though the same ratio of peptide and DNA charges was used to assemble both types of complexes. Dynamic light scattering spectroscopy (DLS) measurements of nanoparticles formulated at a lower concentration (formulation 1) revealed the presence of only one distinctive population of particles with an average diameter of 115 nm (Figure 1; Table S1); furthermore, the measured polydispersity index was rather low, approximately 0.16 (Table S1). Unexpectedly, the *in vivo* formulation (formulation 2) particles contained several populations of nanoparticles with different sizes, and only about 1/3 of these had approximately 100-nm diameter, which is suitable for the *in vivo* application (Figure 1; Table S1). Therefore, particles in formulation 2 have heterogeneous sizes with broad distribution, which can hinder future pharmacological application because among other physiochemical parameters, the particle size and size distribution are essential factors in drug delivery.[9]

Filtration of the Nanocomplex Solution Yields Uniform Nanoparticles

A common approach for eliminating large aggregates and acquiring particles with a uniform size is filtration; therefore, we introduced this additional step, which has been widely applied for the preparation of uniformly sized liposomes.[15] Accordingly, we filtered the solution of our nanoparticles through 100-nm and 200-nm pore-sized filters. As seen in Figure 2 (and Table S1), the standard nanoparticle formulation procedure for *in vivo* use (formulation 2) lead to formation of large conglomerates in solution: 40% of particles were larger than 300 nm, and only about 35% of nanoparticles had an average diameter around 100 nm, whereas the remaining had formed aggregates over 2 μm in size. Furthermore, the polydispersity index was remarkably higher compared to that of formulation 1 (0.61) (Table S1). However, these DLS data should be interpreted with caution because despite DLS being the most commonly used technique for the analysis of nanoparticle size and protein aggregates, it can overestimate the

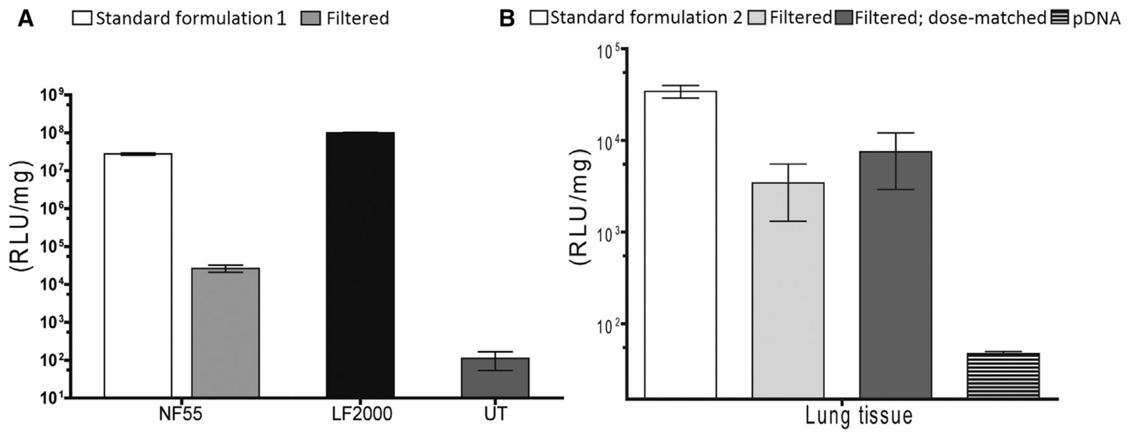

Figure 3. Transfection Efficacy of Filtered NF55/pDNA Nanoparticles

Transfection efficacy of filtered NF55/pDNA nanoparticles in CHO cells at CR4 (A) and in BALB/c mice at CR4 (B). Light gray bars (Filtered) represent filtration results; dark gray bars (Filtered, dose-matched) represent the filtered nanoparticles with matched dose to account for the loss of material during the filtration. Untreated cells (UT) were used as a negative control, and Lipofectamine 2000 (LF2000) was used as a positive control. The data are presented as the mean ± SEM (n = 5).

fraction of larger aggregates, especially in the measurements of sample polydispersity.[16] DLS measurements confirmed that filtration successfully removed large aggregates, and only one distinctive population of about 125-nm nanoparticles was obtained (Figure 2). Furthermore, the polydispersity index was markedly lower compared to that of formulation 2 (0.12) (Table S1). Interestingly, the size-fractionated nanoparticles were stable in time, and even after 60 min of incubation, we could still detect only one population of particles with a defined diameter of 120 nm, as opposed to the formulation 2 particles, which already had a heterogeneous size distribution at the 1-min time point. This indicates that filtration is a beneficial method for increasing the uniformity of the complexes' size.

In order to quantify the loss of nanoparticles upon the filtration step, we analyzed the amount of particles that were recovered after passing the solution through filters. To minimize the adsorption of nanoparticles in the membrane, we chose hydrophilic polypropylene filters that are known to lead to minimal loss of nucleic acid and protein samples. Although the filtration led to some loss of material, still the majority of the peptide (80% and 74%) and plasmid (74% and 60%) were recovered in the filtrate of 200-nm and 100-nm filters, respectively (Figure S1).

Next, we examined whether these stable homogeneous 120-nm nanoparticles that we obtained after filtration also had retained or even had gained the additional bioactivity. Contrary to our expectations, we discovered that the filtered nanoparticles demonstrated a 1,000-fold lower efficacy of transfection of cells in the culture (Figure 3A). Even though some previous studies have shown that the nanoparticle fractions with lower diameter might mostly contribute to the transfection,[12] unfortunately this did not reproduce in our study. Thus, we speculated that in our system the larger nanoparticles might be more efficient in transfecting cell cultures, perhaps due to sedimentation.[13]

Despite the disappointing results in the cell culture, these particles could in principle be still efficient and applicable *in vivo* because the homogeneous size could lead to improved bioactivity and reduced possible side effects. Therefore, we switched to *in vivo* administration of filtered formulation nanoparticles. However, while *standard* nanoparticles of formulation 2 led to high expression of transgenes in lungs and liver, the filtered nanoparticles triggered only a minimal readout in inspected organs (Figures 3B and S2). Even when we matched the doses of nanoparticles to compensate for the loss of particles introduced by filtration, the bioactivity of filtered particles did not markedly improve (Figures 3 and S2). Although the filtration method produced uniform small nanoparticles of appropriate size, these had almost completely lost the biological efficacy. This seemed to indicate that the larger-size fraction of the NF55/DNA nanoparticles is crucial for transfection, and the removal of these minimizes the biological effect.

Cryo-concentrated Nanoparticles Are Uniform and Not Aggregating

Even though filtration yielded stable nanoparticles with homogeneous size distributions, it also decreased the bioactivity. Therefore, we introduced an alternative strategy for producing nanoparticles with a homogeneous and suitable size. We tried to mimic the *in vitro* nanoparticle formulation protocol (formulation 1), which produces particles of rather homogeneous size and narrow distribution, and adapted it for *in vivo* settings. Accordingly, we assembled the complexes at the same concentration of peptide and plasmid as we did for formulation 1, lyophilized the nanoparticles, and reconstituted the sample in a smaller volume to reach high concentration required for administration *in vivo*. These particles are subsequently referred to as cryo-concentrated complexes (CCCs).

Next, we assessed whether freezing, drying, or resuspension of nanoparticles occurring during the formulation procedure affects the size

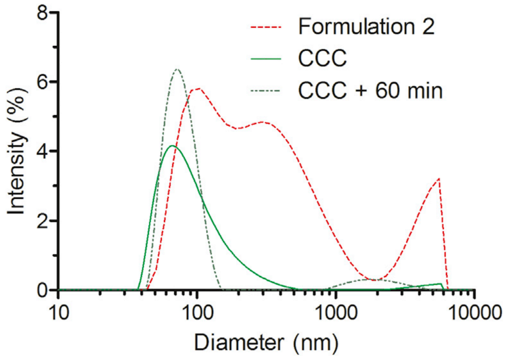

Figure 4. Size Distribution of CCC NF55/DNA Nanoparticles

The particles obtained by complex formulation method 2 are represented with a dashed red line, and the cryo-concentration complex (CCC) formulation strategy is illustrated with a green line. The dotted darker green line shows the size distribution of CCC particles after incubation for 60 min at room temperature. Data were analyzed using the Malvern Zetasizer.

and aggregation of the nanoparticles. According to DLS, CCC nanoparticles demonstrated markedly higher size homogeneity than the formulation 2 ("particles *in vivo*"). About 98% of the CCC nanoparticles had diameter of about 100 nm, and only 2% of the particles had aggregated and formed conglomerates around the micrometer size (Figure 4; Table S1). CCC particles demonstrated better stability in time; even after 60 min of incubation, the nanoparticles remained mostly homogeneous. Approximately 13% of the particles still conglomerated to aggregates with hydrodynamic diameter of approximately 2 μm (Figure 4; Table S1).

Transmission Electron Microscopy Confirms that Cryo-concentrated Complexes Yield Uniform Particles

The differences in the DLS pattern prompted us to analyze the morphology of nanoparticles obtained by using different formulation strategies harnessing electron microscopy resolution. Complexation of pDNA with NF55 at high concentration required for *in vivo* studies (formulation 2) lead to formation of highly diverse nanoparticles that differed both in size and shape when standard formulation strategy was used (Figure 5A). Smaller nanocomplexes of about 60–65 nm in diameter were mostly spherical, whereas larger particles had up to 5-fold higher diameter and irregular shape. The shape of the latter implied association of several smaller nanocomplexes to form a bigger nanoparticle; however, individual smaller nanocomplexes could not be clearly distinguished within the bigger particles. The individual nanoparticles assembled using standard complex formulation strategy showed strong tendencies to associate with each other to form larger conglomerates that might represent the high-sized particles detected by DLS. The large agglomerates of NF55/pDNA complexes can also be removed by the filtration of nanocomplex-containing solution through the nano-porous filters. Indeed, filtration of the formulation 2 solution through the filter with cutoff at 200 nm removed particles exceeding this limit, leaving the majority of nanoparticles in solution (Figure 5B).

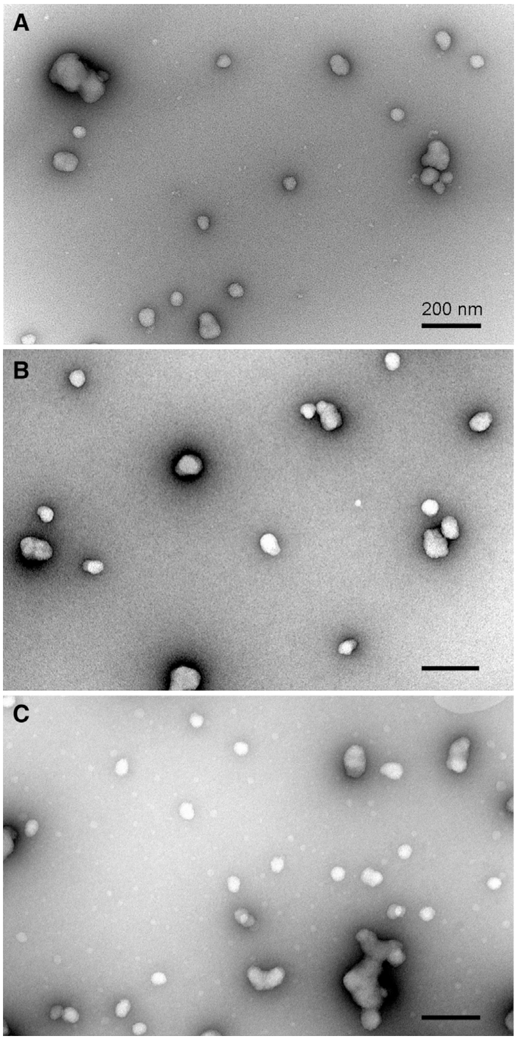

Figure 5. Morphology of NF55/pDNA Nanoparticles Formulated in Different Conditions

The nanoparticles were visualized by negative staining with uranyl acetate and transmission electron microscopy. (A) Formulation 2, (B) filtered formulation 2, (C) cryo-concentrated and reconstituted formulation 1. Scale bars, 200 nm.

An alternative strategy for avoiding the formation of aggregates and bulky particles in NF55/pDNA specimens is to assemble nanocomplexes using lower concentrations of components, similar to the nanoparticles used in tissue culture settings (formulation 1). Freeze drying is known to preserve the morphology of NF55/pDNA nanoparticles, and this also enables reconstitution of nanoparticles at the high concentration that is required for *in vivo* experiments.[4] The specimen of cryo-concentrated NF55/pDNA nanoparticles contains mostly separate spherical particles with about 50 nm diameter and no large irregular structures (Figure 5C). However, the tendency to cluster to assemblages is also inherent for this type of nanoparticle, and often 2–3 particles associate and generate elongated and worm-like structures (Figure 5C). The latter also seem to form upon the

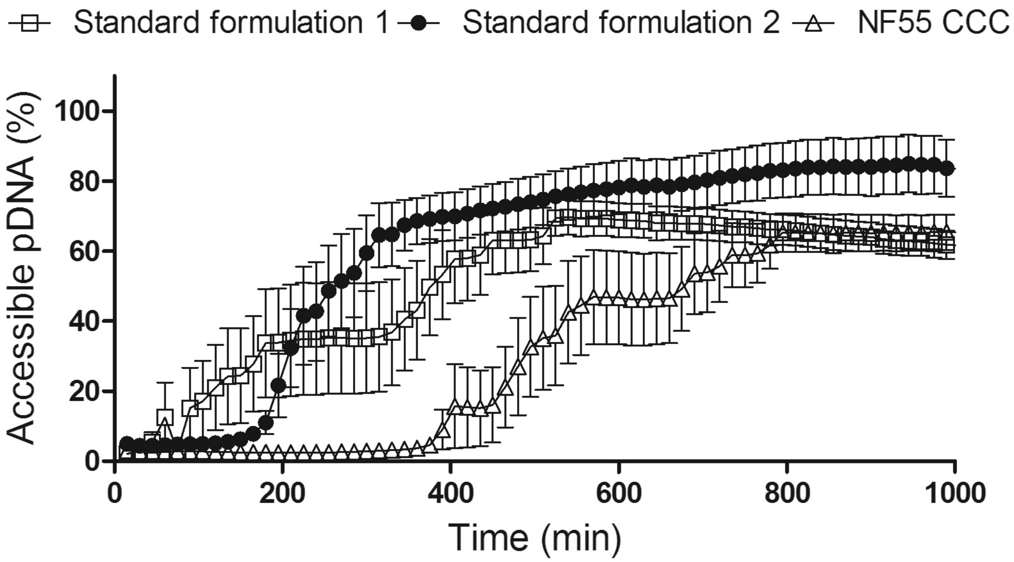

Figure 6. Resistance of NF55/DNA Complexes to Proteinase K Treatment

Resistance to Proteinase K treatment of NF55/DNA complexes formed according to standard formulation methods (formulation 1 and formulation 2) and cryo-concentration (CCC) formulation strategy. The results were normalized to total DNA amount in the complexes.

storage of nanoparticle solution, and accumulation of bigger particles in time was also measured by DLS (Figure 4).

CCC Particles Are Less Susceptible to Enzymatic Degradation

The stability of nanoparticles in the blood stream is crucial for their application in drug delivery *in vivo*. To analyze the stability of CCC particles, we analyzed the resistance of these nanoparticles to protease digestion. The particles acquired by the standard formulation 1 procedure started to release pDNA from the complexes almost immediately, gradually releasing the pDNA over longer periods of time (Figure 6). In contrast, the formulation 2 complexes persisted for about 2 hr before free pDNA started to appear, but once the degradation of the complexes commenced, the process progressed fast. Finally, the CCC particles displayed the highest resistance against the protease, persisting for about 3 hr before the degradation started. This notable stability of the CCC complexes is in accord with our previous report, where we showed that lyophilized NF55/DNA nanoparticles were stable for at least 7 days at room temperature.[4] Therefore, relying on our new and previous results, we suggest that CCC lyophilized particles could be prepared and conveniently stored as solid formulations that retain the homogeneous size until reconstituted in water and administered according to the needs of the particular application. This advantage of solid drug formulation enables easier handling, longer storage, convenient transportation, and potential benefit in reproducibility.

Cryo-concentrated Nanoparticles Have Higher Bioactivity *In Vivo*

After confirming that we have homogeneous and stable nanoparticles, we first assessed the bioactivity of NF55/pDNA CCC in cell cultures. CCC particles exhibited similar transfection efficiency as standard formulation 1 (Figure S3). This encouraged us to study whether the CCC formulation also possesses bioactivity *in vivo*. Surprisingly, cryo-concentrated nanoparticles generated more than 2-fold higher lung transfection compared to complexes prepared with the standard formulation procedure (Figure 7). Histological analysis did not reveal any pathological changes in the lung of CCC-treated animals (Figure S5). In all other analyzed tissues, the expression of reporter gene remained at a similar level with standard formulation 2 (Figure S4). These findings demonstrate that our new CCC formulation strategy generates particles with a uniform size that transfect lung tissue significantly better than the particles prepared using the standard complex formulation method.

This brought us to an intriguing situation: starting from the heterogeneous particle mixture of formulation 2 (Figure 1) and by using two different methods to acquire particles with more homogeneous size distribution (Figures 2 and 4), with one we reduced and with the other improved the efficacy. One may question whether there is a particular size of the particles that mediates transfection and whether the same size suits both the *in vitro* and *in vivo* transfection. The fact that the filtered samples of nanoparticles show reduced bioactivity suggests that the larger conglomerates may facilitate transfection. Indeed, the larger particle size and aggregation have been associated with faster sedimentation and also with the higher uptake in cell cultures.[14] Concerning the *in vivo* experiments, previous results with different types of nanoparticles have indicated that larger particles tend to accumulate within the capillaries of the lungs.[9] These data might help to explain why the NF55/pDNA-filtered fraction that contains only small nanoparticles exhibits reduced bioactivity in lungs. To shed light on the question of if only the size of the particles affects

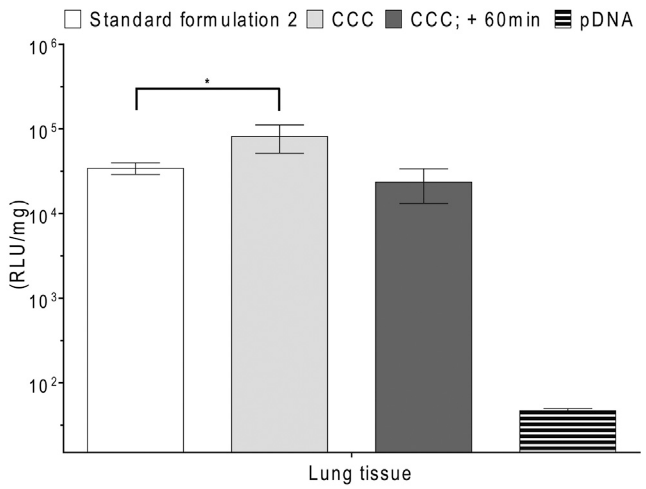

Figure 7. Expression of Marker Gene in BALB/c Mice Transfected by NF55/pDNA Nanoparticles Produced by CCC and Standard Formulation 2 at CR4
The data from at least five representative experiments are presented as the mean ± SEM, $*p < 0.05$ (Student's t test, two-tailed distribution, two-sample unequal variance).

transfection efficacy, we promoted the conglomeration of the CCC particles by incubating these at room temperature for 60 min, instead of using them immediately after the reconstitution of nanocomplexes. DLS shows that incubation of CCC particles for 60 min induces a positive skew to the size distribution, increasing the fraction with larger size (Figure 4). In addition, aggregation is observed in about 13% of the particles (compared to 3% at 1 min) (Figure 4; Table S1). If particles with larger size were to contribute to more efficient transfection, then the CCC after 60 minutes of pre-incubation should promote higher transfection. Interestingly, however, these particles exhibited slightly reduced transfection efficiency, showing the effect at same level with the standard formulation 2 (Figure 7). We may conclude that in the case of NF55/pDNA nanoparticles, the size of particles is not the only factor to be considered, and other parameters, such as the uniformity and stability of the particles, might also modulate their bioactivity.

Taken together, in the current study, we have elaborated a new formulation method for manufacturing uniformly sized and non-aggregating CPP-pDNA nanoparticles. The resistance of particles produced by a cryo-concentration approach to enzymatic degradation was substantially increased compared to particles obtained by standard formulation strategy, encouraging further applications *in vivo*. Importantly, the novel particles also induced significantly higher transfection of reporter genes *in vivo* than standard nanoparticles, confirming their high bioefficacy. The CCC formulation strategy produces efficiently transfecting NF55/pDNA nanoparticles in solid form that allows long-time storage and quick and easy reconstitution at convenient time and at required dose and concentration. We hereby propose a new strategy for producing pDNA transfection particles that remain stable during storage and allow convenient use at required time and guarantee enhanced *in vivo* gene delivery efficacy.

MATERIALS AND METHODS

Peptide Synthesis

Peptides were synthesized using the solidCphase Fmoc peptide synthesis stepwise method, cleaved from resin and purified as described previously.[14]

Formulation of NF55 and DNA complexes

Formulation 1

NF55/pDNA nanoparticles were prepared by mixing 0.5 μg of plasmid (p-CMV-Luc2) with the peptide in a total volume of 50 μL MilliQ (MQ) water. The nanoparticles were allowed to assemble/stabilize at room temperature for 30 min before being used in transfection experiments, and this is a standard formulation for the cell culture transfection experiments. The charge ratio (CR) of 4 (CR, equivalent to the nitrogen-to-phosphate ratio) was used throughout current study, in which case the final peptide concentration was 40 μM.

Formulation 2

Twenty micrograms of p-CMV-Luc2 was mixed with the peptide in a total volume of 150 μL MQ. After 30 min of incubation, glucose was added to a final 5% glucose in 200 μL and used in transfection experiments. This is a standard formulation used for *in vivo* studies, where 20 μg of pDNA is administered per one animal (1 mg/kg dose). Within the current experiments, CR4 was used, in which case the final peptide concentration was 400 μM.

Filtration

For the removal of aggregated particles, complexes were formulated as described above (formulation 2) (except that the initial formulation volume was slightly larger to compensate for the loss of solution remaining in the filter) and then filtered through 100 nm or 200 nm pore-sized polypropylene filters (Pall, Acrodisc, USA). To quantify the recovery of plasmid and peptide after filtration, PicoGreen (Quant-iT PicoGreen, Thermo Fisher Scientific) and the Lowry-based protein detection method (DC protein determination kit, Bio-Rad) were used. For *in vitro* studies, we diluted complexes 10-fold, thereby achieving a concentration equal to formulation 1.

Cryo-concentrated Nanoparticles

To formulate the cryo-concentrated complexes (CCCs), a combination of formulation 1 and 2 was used. Twenty micrograms of p-CMV-Luc2 plasmid was mixed with the peptide at CR4 according to formulation 1 (final peptide concentration 40 μM in a total volume of 2 mL of MQ water). After 30 min of incubation, glucose was added according to formulation 2 (final glucose amount equivalent to 5% glucose in 200 μL), and thereafter, the formulated complexes were lyophilized. Prior to use in transfection experiments, the complexes were reconstituted in 200 μL of MQ water. This recipe represents a dose for one animal (1 mg/kg). For *in vitro* studies, we diluted complexes 10-fold, thereby achieving a concentration equal to formulation 1.

Proteinase K Assay

Proteinase K treatment was carried out to evaluate the stability of the preformed NF/DNA complexes to enzymatic degradation of CPP by proteinases. The preformed complex solution and controls with either pDNA (0.05 μg per sample) or MQ water were transferred to a black 96-well plate, and 90 μL of MQ water was added. After a short period of mixing, a fluorescent DNA intercalating dye dilution was added (Quant-iT PicoGreen, Thermo Fisher Scientific) and incubated for 5 min at room temperature. The fluorescence was measured (Synergy MX, BioTek; λ_{ex} 492 nm and λ_{em} 535 nm) to quantify the initial accessibility of pDNA. Thereafter, 20 μL of Proteinase K (~20 mg/mL, > 600 U/mL, Thermo Scientific) was mixed with 1 mL of MQ water, and 10 μL of this solution was transferred to each well and incubated for 10 min at 26°C. Fluorescence was measured over a period of 16 hr at 26°C. These readings were normalized to free pDNA and MQ fluorescence and expressed as the digestion time required to release 50% of the pDNA from NF55/pDNA complexes.

Cell Culture and Transfection

Chinese hamster ovary (CHO) cells were grown in DMEM, supplemented with GlutaMAX, 0.1 mM non-essential amino acids, 1.0 mM sodium pyruvate, 10% fetal bovine serum (FBS), 100 U/mL penicillin, and 100 mg/mL streptomycin (PAA Laboratories, Germany).

For transfection studies, 50,000 CHO cells were seeded 24 hr before the experiment into 24-well plates, treated with complexes for 4 hr in serum-containing medium, incubated 20 hr, and measured for luminescence as described previously.[4]

In Vivo Studies

Reporter gene expression levels were evaluated post mortem 24 hr after injecting the complexes into the tail vein. We have previously shown that the 24 hr time point is optimal for p-CMV-Luc2.[8] For the *in vivo* studies, male and female BALB/c mice (8 weeks old) were used. Each animal received a single 200-μL intravenous (i.v.) injection of the complexes. After 24 hr, the mice were sacrificed using cervical dislocation, and whole organs were harvested and snap-frozen on dry ice. The organs were homogenized, and luminescence was measured as described previously.[8] For the histological evaluation, the lungs were inflated postmortem with 1:1 cryomatrix:PBS (OCT embedding matrix, Kaltek, Italy), frozen in 2-methylbutane bath on dry ice. Fresh cryosections (10-μm thick) were fixed with formalin and stained with H&E.

All animal experiments and procedures were approved by the Estonian Laboratory Animal Ethics Committee (approvals no 81, dated April 4, 2016, and 69 and 70, dated February 9, 2011).

Dynamic Light Scattering

The mean hydrodynamic diameters of the nanocomplexes were determined by dynamic light scattering studies using a Zetasizer Nano ZS apparatus (Malvern Instruments, United Kingdom). All results were based on three or four measurements from two independent samples. All data were converted to relative by intensity or volume plots from which the mean hydrodynamic diameters were derived.

Transmission Electron Microscopy

For the morphological characterization of complexes, a negative-staining TEM analysis was performed as described previously.[16] In brief, copper grids were covered with a formvar film and a carbon layer using a Leica EM ACE600 carbon coater (Leica Microsystems, Germany). Then, 10-μL aliquots of each sample were applied onto the grids for 1 min, followed by washing with MQ water. Thereafter, samples were exposed to 2% aqueous uranyl acetate solution for 4 min. Next, the excess stain was removed with filter paper and the samples were allowed to air-dry. The specimens were imaged at 120 kV accelerating voltage using an FEI Tecnai G2 Spirit transmission electron microscope (FEI, the Netherlands).

SUPPLEMENTAL INFORMATION

Supplemental Information includes five figures and one table and can be found with this article online at https://doi.org/10.1016/j.omtn.2017.10.011.

AUTHOR CONTRIBUTIONS

P.A., K.F., K.K., L.P., R.R., M.P., and A.P. conducted the experiments; K.F., K.K., and P.A. designed the experiments; and K.F., P.A., K.K., and M.P. wrote the paper; Ü.L. supervised.

CONFLICTS OF INTEREST

The authors have no conflicts of interest to disclose.

ACKNOWLEDGMENTS

The authors thank Kaida Koppel for preparing specimens for TEM and Anu Kõiveer for the preparation of the H&E tissue slides. This work was supported by the European Regional Development Fund through the projects Tumor-Tech (3.2.1001.11-0008) and Center of Excellence in Molecular Cell Engineering (2014-2020.4.01.15-0013) and by the Estonian Ministry of Education and Research (0180019s11) and Estonian Research Council (IUT20-26 and PUT1617).

REFERENCES

1. Yin, H., Kanasty, R.L., Eltoukhy, A.A., Vegas, A.J., Dorkin, J.R., and Anderson, D.G. (2014). Non-viral vectors for gene-based therapy. Nat. Rev. Genet. *15*, 541–555.

2. Wittrup, A., and Lieberman, J. (2015). Knocking down disease: a progress report on siRNA therapeutics. Nat. Rev. Genet. *16*, 543–552.

3. Guidotti, G., Brambilla, L., and Rossi, D. (2017). Cell-penetrating peptides: from basic research to clinics. Trends Pharmacol. Sci. *38*, 406–424.

4. Freimann, K., Arukuusk, P., Kurrikoff, K., Vasconcelos, L.D.F., Veiman, K.-L., Uusna, J., Margus, H., Garcia-Sosa, A.T., Pooga, M., and Langel, Ü. (2016). Optimization of in vivo DNA delivery with NickFect peptide vectors. J. Control. Release *241*, 135–143.

5. Ravi Kumar, M.N.V., Bakowsky, U., and Lehr, C.M. (2004). Preparation and characterization of cationic PLGA nanospheres as DNA carriers. Biomaterials *25*, 1771–1777.

6. Düzgüneş, N., Wilschut, J., Hong, K., Fraley, R., Perry, C., Friend, D.S., James, T.L., and Papahadjopoulos, D. (1983). Physicochemical characterization of large unilamellar phospholipid vesicles prepared by reverse-phase evaporation. Biochim. Biophys. Acta *732*, 289–299.

7. Andaloussi, S.E.L., Lehto, T., Mäger, I., Rosenthal-Aizman, K., Oprea, I.I., Simonson, O.E., Sork, H., Ezzat, K., Copolovici, D.M., Kurrikoff, K., et al. (2011). Design of a peptide-based vector, PepFect6, for efficient delivery of siRNA in cell culture and systemically in vivo. Nucleic Acids Res. *39*, 3972–3987.

8. Veiman, K.-L., Künnapuu, K., Lehto, T., Kiisholts, K., Pärn, K., Langel, Ü., and Kurrikoff, K. (2015). PEG shielded MMP sensitive CPPs for efficient and tumor specific gene delivery in vivo. J. Control. Release *209*, 238–247.

9. Blanco, E., Shen, H., and Ferrari, M. (2015). Principles of nanoparticle design for overcoming biological barriers to drug delivery. Nat. Biotechnol. *33*, 941–951.

10. Chen, L.T., and Weiss, L. (1973). The role of the sinus wall in the passage of erythrocytes through the spleen. Blood *41*, 529–537.

11. Choi, H.S., Liu, W., Misra, P., Tanaka, E., Zimmer, J.P., Itty Ipe, B., Bawendi, M.G., and Frangioni, J.V. (2007). Renal clearance of quantum dots. Nat. Biotechnol. *25*, 1165–1170.

12. Prabha, S., Zhou, W.-Z., Panyam, J., and Labhasetwar, V. (2002). Size-dependency of nanoparticle-mediated gene transfection: studies with fractionated nanoparticles. Int. J. Pharm. *244*, 105–115.

13. Cho, E.C., Zhang, Q., and Xia, Y. (2011). The effect of sedimentation and diffusion on cellular uptake of gold nanoparticles. Nat. Nanotechnol. *6*, 385–391.

14. Arukuusk, P., Pärnaste, L., Oskolkov, N., Copolovici, D.-M., Margus, H., Padari, K., Möll, K., Maslovskaja, J., Tegova, R., Kivi, G., et al. (2013). New generation of efficient peptide-based vectors, NickFects, for the delivery of nucleic acids. Biochim. Biophys. Acta *1828*, 1365–1373.

15. Olson, F., Hunt, C.A., Szoka, F.C., Vail, W.J., and Papahadjopoulos, D. (1979). Preparation of liposomes of defined size distribution by extrusion through polycarbonate membranes. Biochim. Biophys. Acta *557*, 9–23.

16. Margus, H., Arukuusk, P., Langel, Ü., and Pooga, M. (2016). Characteristics of cell-penetrating peptide/nucleic acid nanoparticles. Mol Pharm. *13*, 172–179.

OMTN, Volume *10*

Supplemental Information

Formulation of Stable and Homogeneous Cell-Penetrating Peptide NF55 Nanoparticles for Efficient Gene Delivery *In Vivo*

Krista Freimann, Piret Arukuusk, Kaido Kurrikoff, Ly Pärnaste, Raivo Raid, Andres Piirsoo, Margus Pooga, and Ülo Langel

Supplementary information

Table S1. The mean hydrodynamic diameter of the differently formulated nanoparticles

Formulation of nanoparticles	Time, min	Hydrodynamic diameter, nm (Intensity)	pDI	Size distribution by volume (%)
Formulation 1	1-60	124.8 ± 1.6	0.142 ± 0.01	98.6
Formulation 2	1	112.4 ±6.55<br>423 ± 59.57<br>4295 ± 36.23	0.608 ± 0.024	35<br>40.3<br>24.6
Formulation 2 Filtrated 200 nm	1	108.4 ± 5.39	0.122 ± 0.014	100
Formulation 2 Filtrated 200 nm	60	115 ± 4.285	0.120 ± 0.017	100
CCC	1	85.11 ± 14.02<br>2082 ± 991.6	0.211 ± 0.009	97.3<br>2.7
CCC	60	77.8 ± 0.41<br>2055 ± 818.2	0.184 ± 0.023	87.5<br>12.5

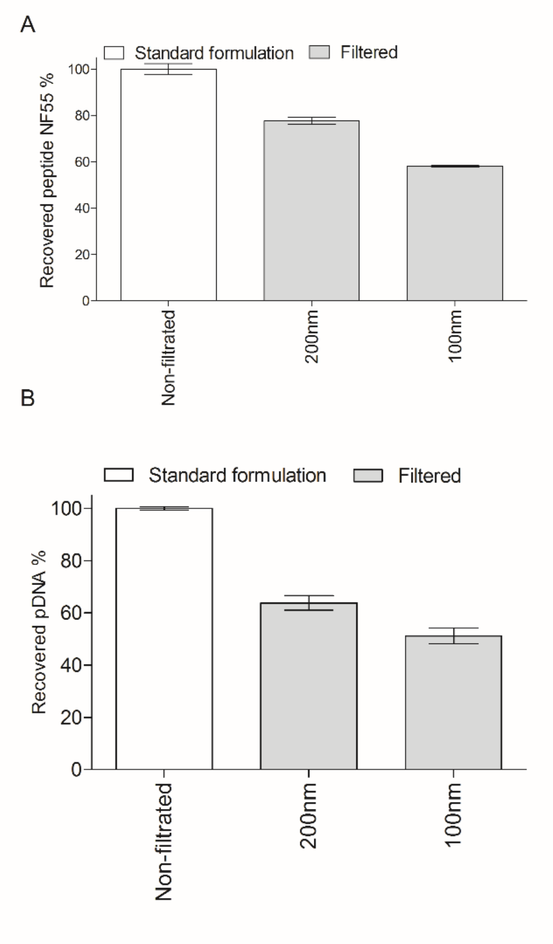

Figure S1. Recovery of peptide (A) and pDNA (B) after filtration of the formulated nanoparticles. PicoGreen (for pDNA) and the DC protein determination kit were used respectively to quantify the components of nanoparticles. The data are presented as the mean ± SEM (*n* = 3)

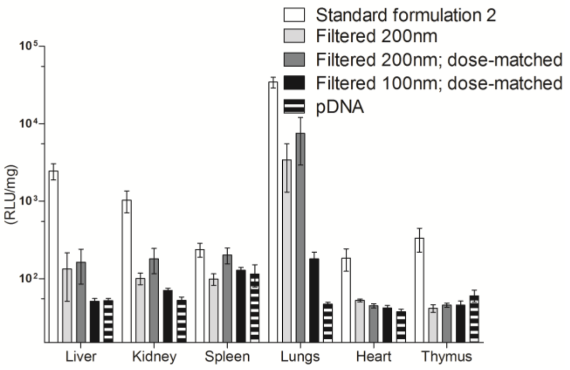

Figure S2. Expression of marker gene in Balb/c mice transfected by NF55/pDNA nanoparticles after filtration at CR4. The data are from at least five representative experiments, presented as the mean ± SEM

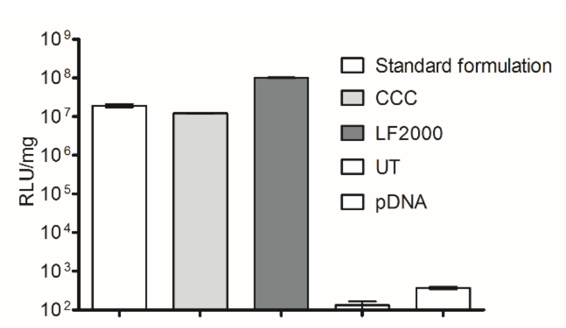

Figure S3. Expression of marker gene in CHO cells transfected by NF55/pDNA CCC nanoparticles at CR4. Untreated cells (UT) were used as a negative control and Lipofectamine™ 2000 (LF2000) was used as a positive control. The data are presented as the mean ± SEM (*n* = 3)

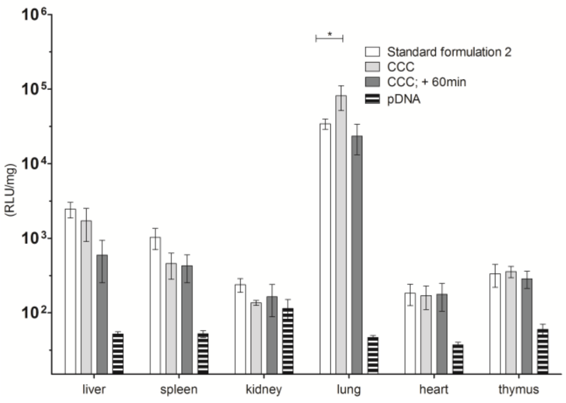

Figure S4. Expression of marker gene in Balb/c mice transfected by NF55/pDNA nanoparticles produced by CCC and standard formulation 2 at CR4. The data from at least five representative experiments are presented as the mean ± SEM, $*P < 0.05$ (Student's *t*-test, two-tailed distribution, two-sample unequal variance).

A

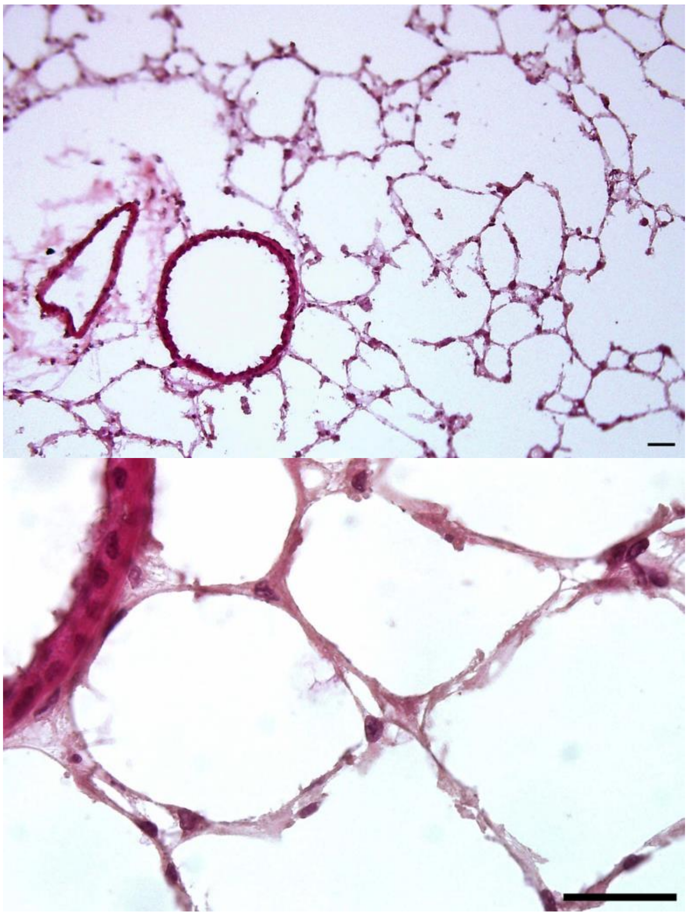

B

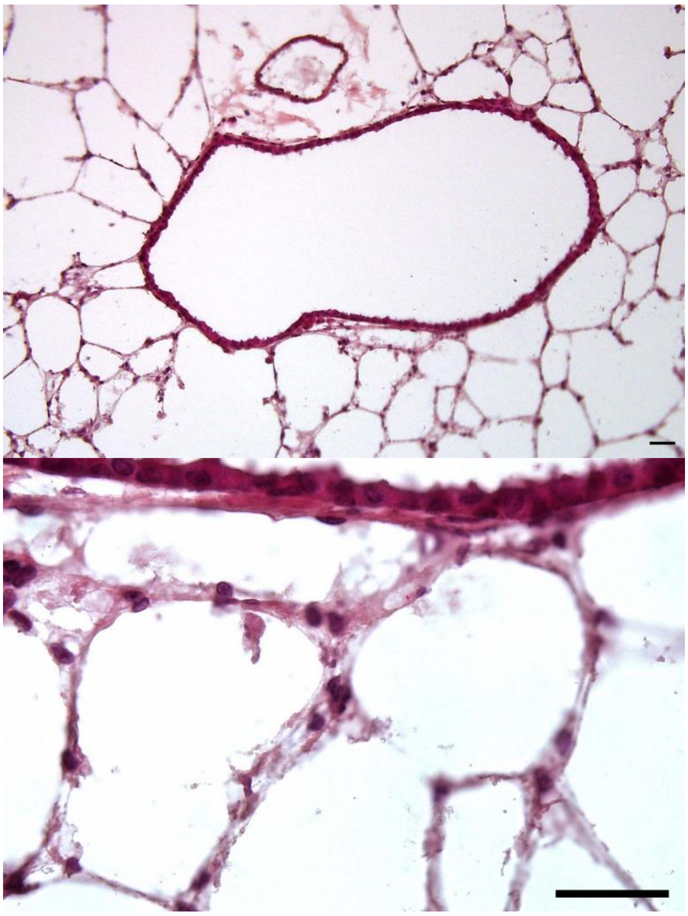

Figure S5. H&E stained tissue from representative lung areas of the animals that have received NF55/pDNA CCC (1 mg/kg pDNA dose) (A) or NF55/pDNA CCC with additional 60 min complex incubation (B). The size bar represents 30 μm.
